# Phosphatidylinositol 3-kinase signaling in proliferating cells maintains an anti-apoptotic transcriptional program mediated by inhibition of FOXO and non-canonical activation of NFκB transcription factors

**DOI:** 10.1186/1471-2121-9-6

**Published:** 2008-01-28

**Authors:** Jolyon Terragni, Julie R Graham, Kenneth W Adams, Michael E Schaffer, John W Tullai, Geoffrey M Cooper

**Affiliations:** 1 Department of Biology, Boston University, Boston MA 02215, USA; 2 Current Address: Alzheimer's Disease Research Center, Massachusetts General Hospital, Charlestown, MA 02129, USA; 3 Current Address: Pfizer, Inc., Research Technology Center, 620 Memorial Drive, Cambridge, MA 02139, USA

## Abstract

**Background:**

Phosphatidylinositol (PI) 3-kinase is activated by a variety of growth factor receptors and the PI 3-kinase/Akt signaling pathway is a key regulator of cell proliferation and survival. The downstream targets of PI 3-kinase/Akt signaling include direct regulators of cell cycle progression and apoptosis as well as a number of transcription factors. Growth factor stimulation of quiescent cells leads to robust activation of PI 3-kinase, induction of immediate-early genes, and re-entry into the cell cycle. A lower level of PI 3-kinase signaling is also required for the proliferation and survival of cells maintained in the presence of growth factors, but the gene expression program controlled by PI 3-kinase signaling in proliferating cells has not been elucidated.

**Results:**

We used microarray analyses to characterize the changes in gene expression resulting from inhibition of PI 3-kinase in proliferating cells. The genes regulated by inhibition of PI 3-kinase in proliferating cells were distinct from genes induced by growth factor stimulation of quiescent cells and highly enriched in genes that regulate programmed cell death. Computational analyses followed by chromatin immunoprecipitations demonstrated FOXO binding to both previously known and novel sites in promoter regions of approximately one-third of the up-regulated genes, consistent with activation of FOXO1 and FOXO3a in response to inhibition of PI 3-kinase. NFκB binding sites were similarly identified in promoter regions of over one-third of the down-regulated genes. RelB was constitutively bound to promoter regions in cells maintained in serum, however binding decreased following PI 3-kinase inhibition, indicating that PI 3-kinase signaling activates NFκB via the non-canonical pathway in proliferating cells. Approximately 70% of the genes targeted by FOXO and NFκB regulate cell proliferation and apoptosis, including several regulators of apoptosis that were not previously known to be targeted by these transcription factors.

**Conclusion:**

PI 3-kinase signaling in proliferating cells regulates a novel transcriptional program that is highly enriched in genes that regulate apoptosis. At least one-third of these genes are regulated either by FOXO transcription factors, which are activated following PI 3-kinase inhibition, or by RelB, which is activated by PI 3-kinase via the non-canonical pathway in proliferating cells.

## Background

The PI 3-kinase/Akt signaling pathway plays a critical role in the regulation of growth factor-dependent metabolism, proliferation and survival of mammalian cells [1,2]. The downstream targets of Akt that function to regulate cell proliferation and survival include the Bcl-2 family member Bad [3,4] and the pro-apoptotic protein kinase GSK-3 [5,6], both of which are inhibited by Akt phosphorylation. Targets of GSK-3 that have been implicated in cell proliferation and survival include the Bcl-2 family member Mcl-1 [7], cyclin D1 [8], and the translation initiation factor eIF2B [9]. In addition, both Akt and GSK-3 phosphorylate a variety of transcription factors [10-13], and transcriptional regulation plays an important role in the control of cell growth and survival by PI 3-kinase/Akt/GSK-3 signaling. For example, the FOXO transcription factors are well-characterized substrates of Akt with key roles in cell proliferation and apoptosis. Phosphorylation by Akt leads to the retention of FOXOs in the cytoplasm as a result of binding to 14-3-3 proteins [14,15]. In the absence of PI 3-kinase/Akt signaling, FOXOs translocate to the nucleus and activate transcription of their target genes, including those that encode proteins that induce cell cycle arrest (e.g., p130, p27 and cyclin G2) and apoptosis (e.g., Fas ligand, Trail, and Bim) [16]. Additional transcription factors that are regulated either directly or indirectly by Akt and/or GSK-3 and may be involved in control of PI 3-kinase-dependent cell proliferation and survival include p53 [17], YAP [18], NFκB [19,20], CREB [21,22], c-Myc [23,24], and c-Jun [25].

Although studies of individual transcription factors and their target genes have elucidated several aspects of PI 3-kinase signaling, understanding the overall program of transcriptional regulation controlled by the PI 3-kinase/Akt/GSK-3 pathway requires global expression analysis. We and others have previously used global expression profiling to identify genes whose induction is dependent on PI 3-kinase signaling following growth factor stimulation of quiescent cells [26-29]. Computational analysis to identify transcription factor binding sites that were over-represented in upstream regions of the PI 3-kinase dependent genes further implicated FOXO, NFκB and CREB as regulators of the induction of these genes in response to growth factor stimulation [26]. Additional studies have identified a subset of these PI 3-kinase-regulated genes that are controlled by GSK-3 and have shown that inhibition of CREB by GSK-3 plays a key role in repressing PI 3-kinase-dependent gene expression in quiescent cells [30].

These studies of transcriptional regulation downstream of PI 3-kinase have examined gene expression in quiescent cells that have been acutely stimulated by growth factor, leading to the robust activation of PI 3-kinase signaling, the rapid induction of immediate-early genes, and the proliferation of cells arrested in G_0_. A lower level of PI 3-kinase signaling is also required for the survival and proliferation of cells that are normally maintained in the presence of growth factors [31]. In the present study, we have examined the gene expression changes that result from inhibition of PI 3-kinase in cells that are actively proliferating in the presence of serum. These experiments identified a novel program of PI 3-kinase-regulated gene expression that is highly enriched in genes that function as regulators of apoptosis and distinct from the program of gene expression induced by growth factor stimulation of quiescent cells. Combined computational and experimental analyses further demonstrated that FOXO and NFκB are major regulators of genes controlled by PI 3-kinase in proliferating cells, and identified new genes with important roles in apoptosis that are targeted by these transcription factors.

## Results

### Identification of Genes Regulated by PI 3-kinase Signaling in Proliferating Cells

**Figure 1:**
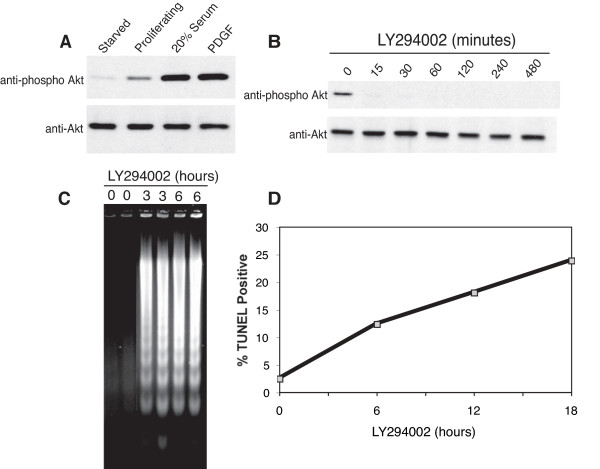
**Effect of PI 3-kinase inhibition on T98G cells**. *A*, Cell extracts were harvested from starved T98G cells that were rendered quiescent by 72 hours of incubation in serum-free medium, from actively proliferating T98G cells in serum-containing medium, and from quiescent cells that had been stimulated by treatment with 20% serum or PDGF for 30 minutes. Extracts were subjected to SDS-PAGE and immunoblotted with anti-phospho Akt and pan anti-Akt antibodies. *B*, Actively proliferating T98G cells in serum-containing media were treated with either 50 μM LY294002 or 0.1% DMSO (vehicle control, 0 minute timepoint). Cell extracts were analyzed by immunoblotting. *C*, Proliferating T98G cells were treated with LY294002 for the indicated times. Cytosolic nucleic acids were isolated and DNA fragmentation was assessed by gel electrophoresis. *D*, Proliferating T98G cells were harvested at the indicated times after LY294002 treatment, subjected to TUNEL assay, and TUNEL-positive cells quantified by flow cytometry.

Global expression changes were analyzed in actively proliferating T98G human glioblastoma cells, which are widely used as a model for studies of growth factor regulation of human cells [26,32,33]. Similar to previous studies in rat fibroblasts [31], these cells maintained a low level of PI 3-kinase signaling, which was 7 fold higher than in quiescent cells but approximately 5-fold lower than that observed following mitogenic stimulation of quiescent cells with either platelet-derived growth factor (PDGF) or 20% serum (Fig. 1A). PI 3-kinase was rapidly inhibited in proliferating cells by addition of LY294002 [34], which inhibited phosphorylation of Akt within 15 minutes (Fig. 1B). Inhibition of PI 3-kinase led to the induction of apoptosis, which initiated as early as 3 hours after addition of LY294002 as indicated by DNA fragmentation (Fig. 1C). The fraction of cells undergoing apoptosis gradually increased over several hours (Fig. 1D), typically reaching levels of 25–50% after 24 hours of PI 3-kinase inhibition.

**Figure 2:**
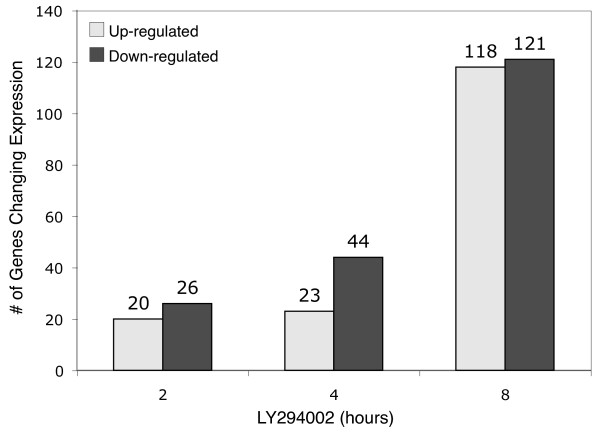
**Changes in gene expression resulting from PI 3-kinase inhibition**. Proliferating T98G cells in serum-containing medium were treated with 50 μM LY294002 for 2, 4 or 8 hours. Microarray analyses at each timepoint were performed on 3 independent cultures compared to untreated controls. Data are presented as the number of genes that were significantly up-regulated and down-regulated at the specified time of treatment (Log_2 _> 0.9, *p *≤ 0.01).

Microarray analyses were performed in three independent experiments to characterize the changes in gene expression resulting from PI 3-kinase inhibition. The number of genes with significantly altered expression (Log_2 _> 0.9; *p *< 0.01) after 2, 4 and 8 hours of treatment with LY294002 are shown in Fig. 2. Inhibition of PI 3-kinase for 2 hours resulted in the up-regulation of 20 genes and down-regulation of 26 genes. After 4 hours of PI 3-kinase inhibition, the number of up-regulated genes slightly increased to a total of 23, whereas the number of down-regulated genes nearly doubled, to a total of 44. The increase in the number of down-regulated genes was most likely due to the slower rates of mRNA degradation for some genes, thus requiring a longer period for significant decreases in mRNA levels to occur. After 8 hours of PI 3-kinase inhibition, the numbers of both up-regulated and down-regulated genes increased substantially to 118 and 121, respectively, possibly resulting from secondary changes in gene expression.

The genes that were up- or down-regulated following 2 and 4 hours of PI 3-kinase inhibition are summarized in Table 1 (see Additional file 1 for expression changes and for genes with altered expression at 8 hours). Most of the up-regulated genes had increased levels of mRNA after both 2 and 4 hours of inhibition, however 4 genes were significantly up-regulated at 2 hours but returned to baseline at 4 hours, perhaps reflecting transient gene induction. Most down-regulated genes displayed reduced mRNA levels at both 2 and 4 hours, generally decreasing at the longer time of PI 3-kinase inhibition. The microarray data for 7 up-regulated and 11 down-regulated genes were validated by real-time RT-PCR. For all genes tested, the expression changes assessed by real-time RT-PCR after 4 hours of PI 3-kinase inhibition confirmed the microarray results (Additional file 1).

**Table 1:** Gene expression changes after 2 and 4 hours of PI 3-kinase inhibition

Up-Regulated Genes		Down-Regulated Genes	
Gene Symbol	Gene Name	Gene Symbol	Gene Name

*APOLD1*	Apolipoprotein L domain containing 1	*AMD1*	S-adenosylmethionine decarboxylase 1
*Atrogin-1*	F-box protein 32/Atrogin-1	*AMIGO2*	Adhesion molecule with Ig-like domain 2
*BCL6*	B-cell CLL/lymphoma 6	*ATP13A3*	ATPase type 13A3
*BTG1*	B-cell translocation gene 1	*BAG2*	BCL2-associated athanogene 2
*C1orf183*	Chromosome 1 open reading frame 183	*BDNF*	Brain-derived neurotrophic factor
*C1orf63*	Chromosome 1 open reading frame 63	*BIRC3/cIAP2*	Baculoviral IAP repeat-containing 3
*CCNG2*	Cyclin G2	*CCL2*	Chemokine (C-C motif) ligand 2
*CLK1*	CDC-like kinase 1	*CCND1*	Cyclin D1
*CTGF*	Connective tissue growth factor	*CYP1B1*	Cytochrome P450 subfamily I polypeptide 1
*DDIT3/CHOP*	DNA-damage-inducible transcript 3	*DCK*	Deoxycytidine kinase
*EIF1/SUI1*	Eukaryotic translation initiation factor 1	*DIO2*	Deiodinase, iodothyronine, type II
*GADD45B*	Growth arrest and DNA-damage-inducible β	*DKFZP686E2158*	Hypothetical protein LOC643155
*ID1*	Inhibitor of DNA binding 1	*DKK1*	Dickkopf homolog 1 (*Xenopus laevis*)
*ID2B*	Striated muscle contraction regulatory prot.	*DSCR1*	Down syndrome critical region gene 1
*IRF7*	Interferon regulatory factor 7	*EDN1*	Endothelin 1
*KIF1B*	Kinesin family member 1B	*F3*	Coagulation factor III
*KLF6*	Kruppel-like factor 6	*FGF2*	Fibroblast growth factor 2
*KLHL24*	Kelch-like 24	*FST*	Follistatin
*LOC85028*	PNAS-123	*G0S2*	Putative lymphocyte G0/G1 switch gene
*MIR*	Myosin regulatory light chain interacting prot.	*GABPB2*	GA binding protein transcription factor, β2
*MT1E*	Metallothionein 1E	*GBP1*	Guanylate binding protein 1
*PCMTD1*	Protein-L-isoaspartate O-methyltransferase	*GBP3*	Guanylate binding protein 3
*PHLDA1*	Pleckstrin homology-like domain family A	*GLIPR1*	Glioma pathogenesis-related protein
*PNPLA8*	Patatin-like phospholipase domain 8	*GPR19*	G protein-coupled receptor 19
*RNU2*	RNA, U2 small nuclear	*GRIK1*	Glutamate receptor, ionotropic, kainate 1
*SAT*	Spermine N1-acetyltransferase	*GRO3*	GRO3 oncogene
*STK31*	Serine/threonine kinase 31	*IGFBP5*	Insulin-like growth factor binding protein 5
*TAF9*	TAF9 RNA polymerase II	*IL7R*	Interleukin 7 receptor
*TIEG*	TGFβ inducible early growth response	*ISG20L1*	Interferon stimulated exonuclease
*TXNIP*	Thioredoxin interacting protein	*LOC57090*	HRPAP20 short form
*ZBTB1*	Zinc finger and BTB domain containing 1	*LTV1*	LTV1 homolog (*S. cerevisiae*)
	cDNA DKFZp564C2063	*MAT2A*	Methionine adenosyltransferase II, alpha
		*MMP3*	Matrix metalloproteinase 3
		*NAV3*	Neuron navigator 3
		*NCOA7*	Nuclear receptor coactivator 7
		*NOLA1*	Nucleolar protein family A, member 1
		*NTF3*	Neurotrophin 3
		*NXT1*	NUTF-like export factor1
		*PAK1IP1*	PAK1 interacting protein 1
		*PLAU*	Plasminogen activator, urokinase
		*PPIF*	Peptidylprolyl isomerase F (cyclophilin F)
		*PTRH2*	Peptidyl-tRNA hydrolase 2
		*PTX3*	Pentaxin-related gene
		*RGS20*	Regulator of G-protein signalling 20
		*SC4MOL*	Sterol-C4-methyl oxidase-like
		*SFRS3*	Splicing factor, arginine/serine-rich 3
		*SGNE1*	Secretory granule, neuroendocrine protein 1
		*SNK*	Serum-inducible kinase
		*STC2*	Stanniocalcin 2
		*TNFRSF11B*	Tumor necrosis factor receptor 11b (OPG)
		*TRIM16*	Tripartite motif-containing 16
		*TSLP*	Thymic stromal lymphopoietin
			cDNA FLJ11812 fis, clone HEMBA1006364

Since LY294002 can also inhibit PI 3-kinase-related protein kinases, such as mTOR, we also tested the effect of wortmannin, a more selective PI 3-kinase inhibitor that does not inhibit mTOR at the concentration used (50 nM) [35]. 15 of the 18 genes were similarly up or down-regulated by both wortmannin and LY294002 (Additional file 2), indicating that PI 3-kinase was responsible for the changes in expression of these genes. It is possible that the 3 genes whose expression was affected by LY294002 but not wortmannin may be regulated by other targets of LY294002. However 50 nM wortmannin was less effective than LY294002 in inhibiting PI 3-kinase, yielding approximately 90% rather than complete inhibition of Akt phosphorylation (Additional file 2), so it is also possible that the 10% of PI 3-kinase signaling remaining after wortmannin treatment was sufficient to maintain normal expression of some genes. In either case, these results support the involvement of PI 3-kinase signaling in the regulation of at least 80–90% of the genes showing altered expression in response to LY294002.

To determine whether gene expression was similarly regulated in a different cell line, the response of representative genes to inhibition of PI 3-kinase was tested in U937 human promyelocytic leukemia cells (Table 2). The changes in expression of 7 out of 12 genes in response to PI 3-kinase inhibition were similar in both U937 and T98G cells, and 3 other genes were either up- or down-regulated, although to different extents, in both cell lines.

**Table 2:** Comparison of the effect of PI 3-kinase inhibition on gene expression in U937 and T98G cells.

	Fold Change	
	U937 cells	T98G cells
**Up-regulated genes**		

*BCL6*	1.3	2.8
***BTG1***	**3.4**	**4.1**
***CCNG2***	**6.6**	**3.0**
*DDIT3/CHOP*	1.3	2.2
***IRF7***	**7.6**	**4.2**
***KLF6/COPEB***	**5.7**	**1.5**

**Down-regulated genes**		

***BAG2***	**3.3**	**4.3**
***BIRC3/cIAP2***	**1.7**	**8.9**
***CCL2***	**51**	**11**
***F3***	**1.8**	**4.8**
***IL7R***	**2.5**	**2.6**
***PLAU***	**3.4**	**2.1**

Proliferating U937 and T98G cells were treated with LY294002 for 4 hours and gene expression was quantitated by RT-PCR. Data are expressed as average fold changes from two independent experiments. Genes in bold were up- or down-regulated in both cell lines.

### Functions of PI 3-kinase-regulated Genes

The genes that showed changes in expression after 2 and 4 hours of PI 3-kinase inhibition were largely overlapping and therefore were combined for subsequent analyses. This yielded a gene set in which altered expression was detected around the onset of apoptosis. These genes were compared to a group of 34 immediate-early genes that were induced by PI 3-kinase signaling following PDGF stimulation of quiescent T98G cells [26]. There were only 4 genes in common between the two groups (*CTGF*, *PLAU*, *CCL2 *and *F3*), indicating that >90% of the genes regulated by continual PI 3-kinase signaling were distinct from immediate-early genes induced by PI 3-kinase in response to acute growth factor stimulation.

The genes with altered expression in response to inhibition of PI 3-kinase in T98G cells for 2 and 4 hours included well-known regulators of apoptosis and cell cycle progression. The ability of PI 3-kinase inhibition to induce apoptosis was apparent in the up-regulation of genes that can promote cell death, including *DDIT3/CHOP *[36], *GADD45B *[37], and *PHLDA1 *[38], and the down-regulation of genes that promote cell survival, including *BIRC3/cIAP2 *[39] and *TNFRSF11B/osteoprotegerin *[40]. Additional expression changes included the up-regulation of genes that inhibit cell cycle progression, such as *CCNG2 *[41], *BTG1 *[42] and *GADD45B *[43], as well as the down-regulation of *CCND1*. Genes encoding several growth factors and cytokines that promote cell proliferation and survival (including *FGF2, NTF3, EDN1*, *CCL2 *and *BDNF*) were also down-regulated following inhibition of PI 3-kinase.

The Gene Ontology (GO) database was used to further characterize the functions of the genes regulated by PI 3-kinase. The frequencies of the GO terms assigned to the PI 3-kinase-regulated set of genes were compared to the frequencies of all annotated genes on the microarray. Biological Process GO terms that were significantly enriched (*p *< 0.001) and identified in at least 15% of the GO-annotated genes that were either up- or down-regulated following 2 and 4 hours of inhibition of PI 3-kinase are indicated in Table 3. The larger set of genes with altered expression after 8 hours of PI 3-kinase inhibition did not display enrichment of GO terms at this level of statistical significance. The up-regulated genes were enriched in GO categories related to development, differentiation, and response to stress, as well as in GO terms related to transcriptional regulation. These GO categories, particularly "response to stress", include such genes as *DDIT3/CHOP *[36] and *GADD45B *[37], and are consistent with the increased expression of genes involved in apoptosis and cell cycle arrest following inhibition of PI 3-kinase. The down-regulated genes were enriched in GO terms related to cell signaling and programmed cell death, consistent with the role of PI 3-kinase signaling in preventing apoptosis. These included genes encoding growth factors and cytokines as well as central negative regulators of apoptosis, such as *BIRC3/cIAP2 *[39] and the TRAIL decoy receptor, *TNFRSF11B*/*osteoprotogerin *[40].

**Table 3:** Summary of Gene Ontology (GO) term analysis.

GO ID	GO name	# Genes	% Genes	*p*-value
**Up-regulated Genes**				

GO:0032502	Developmental process	15	60	0.00002
GO:0048869	Cellular developmental process	12	48	0.00003
GO:0030154	Cell differentiation	12	48	0.00003
GO:0000122	Negative regulation of transcription from RNA polymerase II promoter	4	16	0.00007
GO:0045892	Negative regulation of transcription, DNA-dependent	4	16	0.00033
GO:0006366	Transcription from RNA polymerase II promoter	6	24	0.00064
GO:0006950	Response to stress	7	28	0.00068
GO:0050794	Regulation of cellular process	14	56	0.00087

**Down-regulated Genes**				

GO:0048519	Negative regulation of biological process	13	27	0.000040
GO:0007267	Cell-cell signaling	10	21	0.000040
GO:0007154	Cell communication	23	48	0.000077
GO:0012501	Programmed cell death	10	21	0.000083
GO:0048523	Negative regulation of cellular process	12	25	0.000085
GO:0007165	Signal transduction	21	44	0.000130
GO:0008219	Cell death	10	21	0.000134
GO:0016265	Death	10	21	0.000134
GO:0009887	Organ morphogenesis	7	15	0.000169
GO:0009605	Response to external stimulus	8	17	0.000432
GO:0042221	Response to chemical stimulus	7	15	0.000490
GO:0007166	Cell surface receptor linked signal transduction	12	25	0.000632
GO:0009653	Anatomical structure morphogenesis	10	21	0.000840

All GO terms in the Biological Process category that were significantly enriched [*p *≤ 0.001, Fisher's exact test [76]] amongst the genes changing expression after 2 and 4 hours of LY294002 treatment and were represented in at least 15% of the genes containing GO definitions are listed. The GO ID and name are shown along with the number and percentage of genes that are assigned this term.

### Prediction of Transcription Factor Binding Sites in PI 3-Kinase Regulated Genes

Co-expressed genes are often regulated by common transcription factors, therefore we analyzed the upstream regions of different sets of PI 3-kinase-regulated genes to identify over-represented transcription factor binding sites [26,30,44]. Predictions of functional transcription factor binding sites can be improved by incorporating phylogenetic data to identify sites that are evolutionarily conserved [45], so we focused on sites that were conserved in orthologous genomic regions of human and mouse. We independently analyzed the sets of genes that were either up- or down-regulated following PI 3-kinase inhibition, and also separately analyzed the genes that showed altered expression after 2 and 4 hours of PI 3-kinase inhibition and those that showed altered expression after 8 hours of PI 3-kinase inhibition. Regions 3 kb upstream of the transcription start sites in the human and corresponding mouse sequences were analyzed with the Match program using the minSUM threshold, and both the 546 vertebrate matrices in TRANSFAC Professional v8.4 and the 588 matrices in TRANSFAC Professional v11.1. Over-representation of transcription factor binding sites was assessed by comparing the frequency of predicted sites in each set of genes to the frequencies in a background set of genes that were expressed in proliferating T98G cells but not affected by PI 3-kinase inhibition (see Additional file 3 for complete results).

No significantly over-represented binding sites were identified in the sets of genes that were either up- or down-regulated following 8 hours of PI 3-kinase inhibition, perhaps because these large sets of genes are regulated by a diverse group of transcription factors or by other mechanisms, such as mRNA degradation. However, over-represented binding site matrices were identified in the sets of genes that were either up- or down-regulated after 2 and 4 hours of PI 3-kinase inhibition.

When the results from TRANSFAC v8.4 and v11.1 were combined, there were 9 matrices that were significantly over-represented in the up-regulated genes (Table 4). These included matrices for the forkhead family of transcription factors, as well as for Myc/Max and NF-Y. The most significant matrices represented forkhead family proteins, which together predicted binding sites in 82% of the up-regulated genes. The down-regulated genes were enriched in 4 matrices, 2 for NFκB and 2 for HMGI(Y). When the results from TRANSFAC v8.4 and v11.1 were combined, 27% of the genes had at least one NFκB binding site. The clear enrichment of forkhead and NFκB binding sites in the up- and down-regulated genes, respectively, as well as the fact that members of both of these families of transcription factors are known targets of Akt, led us to further investigate the roles of forkhead and NFκB in the transcriptional response of genes regulated by inhibition of PI 3-kinase.

**Table 4:** Over-represented transcription factor binding sites in genes that were differentially expressed after PI 3-kinase inhibition.

Transcription factor	TRANSFAC Matrix	TRANSFAC version	% Genes with site	*p*-value
**Up-regulated genes**				

Forkhead family members	V$FOXJ2_01	8.4	29	0.004
	V$HFH3_01	8.4	29	0.004
	V$FOXO1_01	8.4	46	0.007
	V$FOXO4_01	8.4	43	0.018
	V$HFH1_01	11.1	57	0.001
	V$FOXO1_01	11.1	71	0.004
	V$FOXO3_01	11.1	75	0.004
	V$FOXO1_02	11.1	71	0.006
	V$HFH8_01	11.1	57	0.009
	V$FOXJ2_01	11.1	43	0.016

Myc/Max	V$MYCMAX_B	8.4	39	0.013
	V$MYCMAX_B	11.1	39	0.009

NF-Y	V$NFY_Q6	11.1	46	0.010

**Down-regulated genes**				

NFκB	V$NFKAPPAB65_01	8.4	12	0.017
	V$NFKAPPAB_01	11.1	27	0.014

HMGI(Y)	V$HMGIY_Q6	11.1	80	0.014
	V$HMGIY_Q3	11.1	73	0.014

The 3 kb regions upstream of the transcription start sites in the human and mouse orthologous sequences were analyzed with the Match algorithm using the MinSUM threshold and both TRANSFAC v8.4 and v11.1 in order to identify over-represented matrices. *P*-values were calculated using the permutation test and adjusted for multiple testing by FDR correction. Significant matrices were those with *p *< 0.02 that predicted less than 1 match in 1 kb of sequence in the background set.

### Analysis of FOXO Regulation and Target Genes

**Figure 3:**
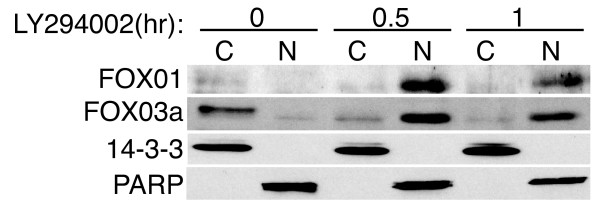
**Nuclear translocation of FOX01 and FOX03a upon LY294002 treatment**. T98G cells were fractionated into nuclear (N; ~2 μg protein) and cytosolic (C; ~25 μg protein) fractions following treatment with LY294002 for the indicated times. Samples were then immunoblotted for the presence of FOX01 and FOX03a. To confirm isolation of nuclear and cytosolic fractions, blots were reprobed with antibodies against PARP (nuclear marker) and 14-3-3 (cytosolic marker). The LY294002 vehicle (DMSO) showed no translocation (data not shown). Shown are representative blots from two independent experiments.

The FOXO transcription factors are inhibited by Akt phosphorylation of Thr-24, Ser-256, and Ser-319 on FOXO1 and the equivalent sites on FOXO3 and FOXO4 [16]. Phosphorylation of these residues results in the binding of 14-3-3 proteins, which inhibits nuclear shuttling and transcriptional activity of the FOXOs. We investigated the expression of FOXO transcription factors in T98G cells and their response to PI 3-kinase inhibition by subcellular fractionation and immunoblotting (Fig. 3). FOXO1 and FOXO3a were expressed in T98G cells and were principally localized to the cytoplasm in cells maintained in serum (FOXO4 was not detectable, data not shown). Addition of LY294002 resulted in the nuclear accumulation of both FOXO1 and FOXO3a within 30 minutes, consistent with their involvement in the transcriptional response of T98G cells to PI 3-kinase inhibition.

**Figure 4:**
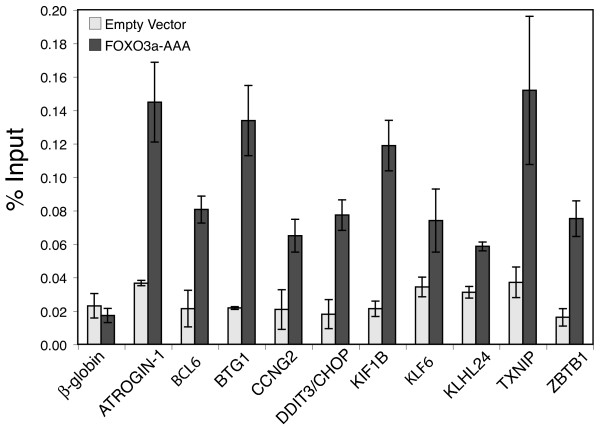
**Analysis of FOXO binding sites by chromatin immunoprecipitation**. T98G cells were transfected with an empty pcDNA3 control or Flag-FOXO3a-AAA expression vector for 24 hours. Chromatin fragments were immunoprecipitated with anti-Flag antibody and quantified by real-time PCR (see Additional file 4 for primers). Data are presented as percentage of input and are the means of 3 independent transfections ± S.E β-*globin *is a negative control. Immunoblots indicated that the expression of FOXO3a-AAA in transfected cells was 3–5 fold greater than the endogenous protein.

Chromatin immunoprecipitation (ChIP) assays were therefore used to test the predicted FOXO binding sites in genes that were induced upon PI 3-kinase inhibition. Exponentially growing T98G cells were transiently transfected with a plasmid expressing a Flag-tagged FOXO3a-AAA mutant [46] and immunoprecipitations of sheared chromatin were performed with Flag-antibody. The Flag-FOXO3a-AAA is a constitutively active mutant in which the three Akt phosphorylation sites have been mutated to alanines, resulting in its constitutive nuclear localization. ChIP assays were performed on three independent transfected cell cultures to test predicted FOXO binding sites upstream of 21 up-regulated genes (two open reading frames with predicted FOXO binding sites were not tested, *C1orf63 *and *C1orf183*, due to their hypothetical protein designation). Flag-FOXO3a-AAA specifically bound to upstream regions of 10 of these 21 genes, compared to cells transfected with empty vector or to the negative control gene *β-globin *(Fig. 4). These verified FOXO binding sites are indicated in Table 5 (see Additional file 4 for all sites tested). These 10 genes included 4 with previously established FOXO upstream binding sites [*CCNG2 *[46,47], *BTG1 *[48], *BCL-6 *[49,50] and *ATROGIN*-*1 *[51] as well as 6 genes not previously identified as having binding sites for FOXO (*ZBTB1*, *KIF1B*, *KLF6*, *KLHL24*, *DDIT3/CHOP *and *TXNIP*).

**Table 5:** FOXO binding sites confirmed by chromatin immunoprecipitation.

Gene	Binding site position	TRANSFAC 8.4	TRANSFAC 11.1	Primer Position
*ATROGIN-1*	-31		V$HFH8_01	-160
	-118	V$FOXO1_01	V$HFH1_01	
	-136	V$FOXO1_01	V$FOXO1_01	

*BCL6*	-837		V$HFH1_01	-1126
	-881		V$FOXO1_02	
	-991	V$FOXJ2_01	V$HFH1_01	
	-1419		V$HFH1_01	

*BTG1*	-213	V$FOXJ2_01	V$FOXO1_01	-254
	-220		V$HFH1_01	
	-496	V$FOXO1_01	V$HFH1_01	

*CCNG2*	-158	V$FOXO1_01	V$HFH1_01	-243
	-165		V$FOXO1_01	
	-469		V$FOXO1_01	
	-473	V$FOXO1_01	V$FOXO3_01	
	-483		V$FOXO1_01	

*DDIT3/CHOP*	-1544		V$FOXO1_01	-1973
	-1606		V$HFH1_01	
	-1830		V$FOXO1_02	
	-2069		V$FOXO1_01	

*K1F1B*	-673		V$FOXO1_01	-673

*KLF6*	-955	V$FOXO1_01	V$HFH1_01	-947
	-929	V$FOXO3_01		
	-887	V$FOXJ2_01	V$HFH1_01	

*KLHL24*	-14		V$FOXO3_01	-211
	-34		V$FOXO1_01	

*TXNIP*	-216	V$FOXO1_01	V$HFH1_01	-250

*ZBTB1*	-252		V$FOXO1_02	-263

The predicted FOXO binding sites that were confirmed by ChIP are indicated by the most 5' nucleotide. When multiple matrices predicted overlapping sites, the position indicates the most 5' nucleotide of the consensus sequence (see Additional file 4 for all sequences). The TRANSFAC matrices shown are those that predicted the indicated sites with the lowest *p*-values. ChIP assays were considered to confirm binding sites located within 500 bp of the primer position. Primer positions are indicated by the most 5' nucleotide of the forward primer; all amplicons were 50–60 bp.

### Analysis of NFκB Regulation and Target Genes

In contrast to the forkhead family, Akt indirectly activates NFκB transcription factors, at least in part by phosphorylation of IκB kinase (IKK) [19,20]. There are 5 different NFκB family members (p65, c-Rel, RelB, p50 and p52), which all bind to a similar consensus sequence and can be activated by several pathways that converge on IKK [52]. Because the majority of NFκB binding sites are occupied after stimulation with cytokines [53], we initially performed ChIP assays following treatment of T98G cells with tumor necrosis factor α (TNFα) to determine if p65 was able to bind to the sites predicted in the genes that were down-regulated upon PI 3-kinase inhibition. In addition to the sites predicted by the matrices indicated in Table 4, we tested conserved sites predicted by 7 additional NFκB matrices to ensure that most functional binding sites were identified. When all matrices were considered, 25 NFκB sites were predicted in a total of 20 of the down-regulated genes (See Additional file 4 for all sites).

**Figure 5:**
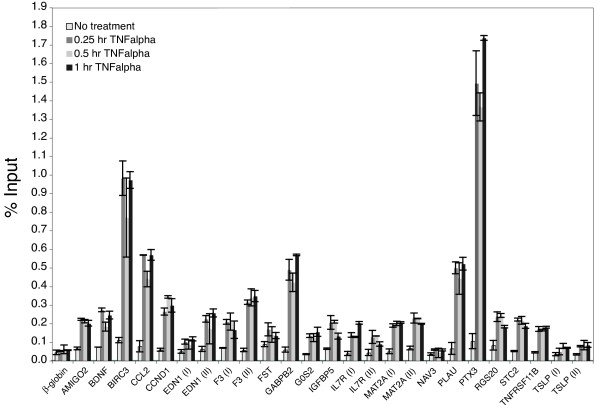
**Analysis of NFκB binding sites in TNFα-stimulated cells by chromatin immunoprecipitation**. T98G cells proliferating in serum-containing media were either left untreated or stimulated with TNFα for 15, 30 or 60 minutes. Chromatin fragments were immunoprecipitated with anti-p65 antibody and quantified by real-time PCR. Data are presented as the percentage of input and are the means of 2 independent experiments ± S.E. β-*globin *was used as the negative control.

TNFα activates NFκB through the canonical pathway, in which IKKβ phosphorylates IκB, which predominantly serves to activate the p50–p65 NFκB heterodimer. Cells treated with TNFα for 0.25, 0.5 or 1 hour were therefore analyzed by ChIP using anti-p65 antibody. Of the 25 predicted sites, 22 sites in 18 genes exhibited increased p65 binding in response to TNFα, which was significantly greater than binding to the β-*globin *negative control (Fig. 5). The binding sites (Table 6) included several that were previously known [*BIRC3/cIAP2 *[54], *CCL2 *[55], *CCND1 *[56], *EDN1 *site I [57], *F3 *site II [58], *GABPB2 *[53], *IL7R *site II [59], *MAT2A *site II [60], *PLAU *[61] and *PTX3 *[62], in addition to novel binding sites in genes known to be targeted by NFκB [*BDNF *[63], *EDN1 *site II, *F3 *site I, *IL7R *site I and *MAT2A *site I], as well as binding sites in novel NFκB-regulated genes (*AMIGO2*, *FST*, *G0S2*, *IGFBP5*, *RGS20*, *STC2 *and *TNFRSF11B/osteoprotegerin*). Note that some of these verified NFκB binding sites were predicted with either Transfac v8.4 or v11.1, but not both.

**Table 6:** NFκB binding sites confirmed by chromatin immunoprecipitation.

Gene	Binding site position	TRANSFAC 8.4	TRANSFAC 11.1	Primer Position
*AMIGO2*	-721		V$NFKAPPAB_01	-795

*BDNF*	-223	V$CREL_01	V$NFKAPPAB_01	-586

*BIRC3/cIAP2*	-92	V$CREL_01		-75
	-115		V$NFKAPPAB65_01	
	-178	V$NFKAPPAB65_01	V$NFKAPPAB_01	

*CCL2*	-2602	V$NFKAPPAB65_01	V$NFKAPPAB_01	-2763
	-2631	V$NFKAPPAB_01	V$NFKAPPAB_01	
	-2804	V$CREL_01	V$NFKAPPAB_01	

*CCND1*	-698	V$CREL_01		-701

*EDN1*	-225 (II)	V$CREL_01		-241
	-293 (II)	V$CREL_01		
	-2263 (I)	V$CREL_01		-2252

*F3*	-188 (II)	V$NFKAPPAB65_01	V$CREL_01	-351
	-482 (II)	V$CREL_01	V$NFKAPPAB_01	
	-2373 (I)	V$NFKAPPAB65_01	V$NFKAPPAB_01	-2358

*FST*	-1335	V$CREL_01		-1341

*GABPB2*	-13	V$CREL_01		-145

*G0S2*	-1832	V$CREL_01		-1914

*IGFBP5*	-176		V$NFKAPPAB_01	-135

*IL7R*	-184 (II)		V$NFKB_C	-184
	-2348 (I)	V$NFKAPPAB65_01	V$NFKAPPAB_01	-2344

*MAT2A*	-343 (II)		V$NFKAPPAB_01	-312
	-1249 (I)	V$CREL_01		-1248

*PLAU*	-1598	V$CREL_01		-1942
	-1838		V$NFKAPPAB65_01	
	-1872	V$NFKAPPAB65_01	V$NFKAPPAB_01	

*PTX3*	-64	V$NFKAPPAB_01	V$NFKAPPAB_01	-147

*RGS20*	-179		V$NFKAPPAB_01	-345

*STC2*	-642		V$NFKAPPAB65_01	-587

*TNFRSF11B/osteoprotegerin*	-128	V$CREL_01		-258

The predicted NFκB binding sites that were confirmed by chromatin immunoprecipitation are indicated by the most 5' nucleotide. When multiple matrices predicted overlapping sites, the position indicates the most 5' nucleotide of the consensus sequence (see Additional file 4 for all sequences). Both over-represented (Table 4) and 7 additional (Additional file 4) NFκB matrices were used for the predictions. The TRANSFAC matrices shown are those that predicted the indicated sites with the lowest *p*-values. ChIP assays were considered to confirm binding sites located within 500 bp of the primer position. The site in the promoter region of *GABPB2 *was predicted using the alignment of the hg17 and mm5 genomes. Multiple binding sites for a gene are designated I and II, where appropriate. Primer positions are indicated by the most 5' nucleotide of the forward primer; all amplicons were 50–55 bp.

p65 did not show increased binding to any of the predicted sites in the absence of TNFα stimulation as compared to the *β-globin *control (Fig. 5), which was expected due to its sequestration in the cytoplasm by IκB proteins. However, other members of the NFκB family can be found in the nucleus of proliferating cells in the absence of cytokine stimulation. p50 and p52 do not bind IκB proteins but rather enter the nucleus following their generation from cleavage of larger precursors (p105 and p100, respectively). Both p50 and p52 lack activation domains, but p52-RelB heterodimers are also able to translocate into the nucleus and activate transcription of their target genes. In the non-canonical pathway of NFκB activation, IKKα phosphorylation of p100 results in its conversion to p52, which forms a transcriptionally active complex with RelB [52]. Since the non-canonical pathway is stimulated by Akt and can be active in the absence of cytokine stimulation [64], it might be responsible for NFκB activity in cells proliferating in serum growth factors.

**Figure 6:**
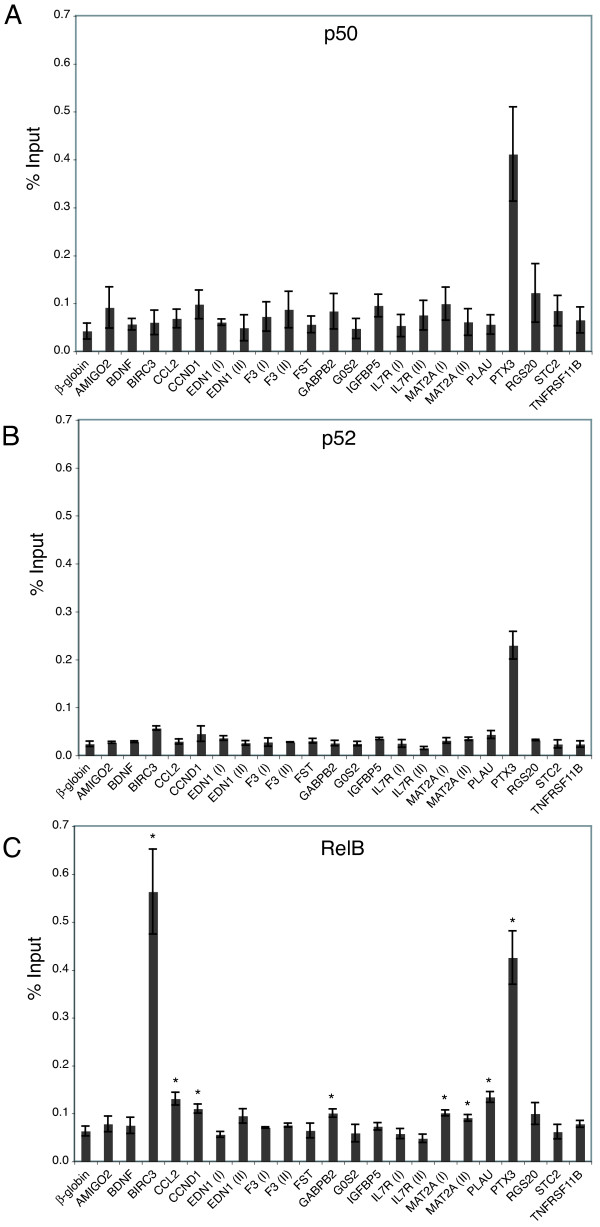
**Analysis of NFκB binding sites in proliferating cells by chromatin immunoprecipitation**. Chromatin fragments from proliferating T98G cells were immunoprecipitated with either anti-p50 (*A*), anti-p52 (*B*), or anti-RelB (*C*) antibody and quantified by real-time PCR. Data are presented as the percentage of input and are the means of 2 independent experiments with anti-p50 and anti-p52 or 3 independent experiments with anti-RelB ± S.E. β-*globin *was used as the negative control. In panel *C*, (*) represents statistically significant binding compared to *β-globin *(assessed by *t*-test).

We therefore performed ChIP assays to measure the basal levels of binding of several NFκB family members to the predicted NFκB binding sites in proliferating cells in the presence of serum. As was observed for p65 (see Fig. 5), c-Rel, which is also activated by the canonical pathway, did not bind to upstream regions of any of the genes (data not shown). In contrast, p50, p52 and RelB bound to the predicted sites in several genes, most strikingly *PTX3 *and *BIRC3/cIAP2 *(Fig. 6). RelB ChIP assays were performed on three independent cultures so that a statistical comparison could be made between the genes with predicted sites and the β-*globin *negative control. This analysis indicated significant binding (*p *< 0.05) of RelB to NFκB sites upstream of 7 genes in T98G cells maintained in serum, including known NFκB sites upstream of *BIRC3/cIAP2*, *CCL2*, *CCND1*, *GABPB2*, *MAT2A *(site II), *PLAU *and *PTX3*, and a novel site in *MAT2A *(site I).

**Figure 7:**
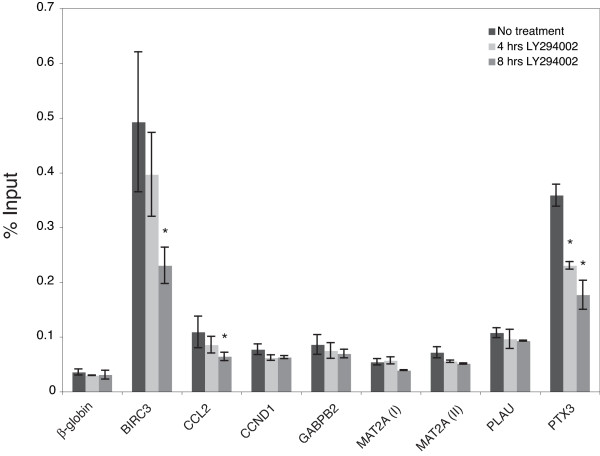
**PI 3-kinase inhibition causes a decrease in RelB binding**. T98G cells were either maintained in serum or treated with 50 μM LY294002 for 4 or 8 hours. Chromatin fragments were immunoprecipitated with anti-RelB antibody and quantified by real-time PCR. Data are presented as the percentage of input and are the means of 3 independent experiments ± S.E. β-*globin *was used as the negative control. (*) represents statistically significant loss of binding compared to the cells maintained in serum (assessed by *t *test).

Because RelB has a transactivation domain and the predicted sites occurred upstream of genes that were down-regulated in response to PI 3-kinase inhibition, we next sought to determine if RelB binding decreased in cells that were treated with LY294002 to inhibit PI 3-kinase. ChIP assays were performed in triplicate to measure RelB binding to the confirmed binding sites in the 7 genes mentioned previously (Fig. 7). Inhibition of PI 3-kinase led to a significant decrease in RelB binding (*p *< 0.05) to the sites in *BIRC3*, *CCL2 *and *PTX3*, consistent with PI 3-kinase regulation of these genes through the non-canonical NFκB pathway.

## Discussion

The PI 3-kinase/Akt/GSK-3 signaling pathway plays a central role in regulation of growth factor-dependent proliferation and survival of mammalian cells, at least in part by transcriptional regulation. A number of transcription factors and target genes that are regulated by PI 3-kinase signaling have been studied on an individual basis. In addition, both we [26,30,44] and others [27-29] have used global expression profiling to investigate the overall program of gene regulation controlled by PI 3-kinase/Akt/GSK-3 signaling. These studies have focused on the response of quiescent cells to growth factor stimulation, which induces robust activation of PI 3-kinase, induction of immediate-early genes, and re-entry into the cell cycle. However, a lower level of continuous PI 3-kinase signaling is also required to maintain proliferation and survival of cells in the presence of serum growth factors. In the present study, we have therefore undertaken a global analysis of the transcriptional program that maintains cell survival and proliferation downstream of PI 3-kinase signaling in proliferating cells.

Inhibition of PI 3-kinase in actively proliferating T98G cells for 2 and 4 hours resulted in up-regulation of 32 genes and down-regulation of 53 genes. These gene expression changes occurred around the time at which apoptosis was first detected. Consistent with their involvement in apoptosis, the genes with altered expression following 2 and 4 hours of PI 3-kinase inhibition included growth factors and cytokines, as well as several well-known regulators of apoptosis (such as *DDIT3/CHOP*, *GADD45B*, *PHLDA1*, *BIRC3/cIAP2 *and *TNFRSF11B/osteoprotegerin*) and cell cycle progression (such as *CCNG2 *and *CCND1*). Functional classifications using the Gene Ontology database further indicated that these gene sets were enriched in terms related to cell stress and programmed cell death, as well as to cell signaling and transcriptional regulation. The numbers of up- and down-regulated genes both increased substantially, to a total of nearly 250 genes, after 8 hours of PI 3-kinase inhibition, probably reflecting a secondary transcriptional response. This larger set of genes was no longer significantly enriched in functional GO terms.

We compared the sets of genes with altered expression following 2 and 4 hours of PI 3-kinase inhibition to those identified by previous studies of the genes induced following 0.5–4 hours of growth factor simulation of quiescent T98G cells [26,44]. Only ~10% of the genes affected by inhibition of PI 3-kinase overlapped with either the total sets of immediate-early or delayed primary response genes, or with the subset of PI 3-kinase dependent immediate-early genes defined in these previous studies. Similar to the genes that were up-regulated following inhibition of PI 3-kinase, immediate-early genes induced in response to growth factor stimulation were enriched in GO terms related to transcriptional regulation [44]. However, the transcription factors induced as immediate-early genes by growth factor stimulation are different from those that are up-regulated in response to PI 3-kinase inhibition. The genes regulated by continuous PI 3-kinase signaling in proliferating cells were thus clearly distinct from the primary response genes induced by growth factor stimulation of quiescent cells.

We also compared the genes affected by PI 3-kinase inhibition with the genes identified in a recent study of the effects of mitogen withdrawal on gene expression in proliferating human fibroblasts [65]. In these experiments, primary fetal human lung fibroblasts were cultured in the presence of a low concentration of serum, IGF-1 and PDGF, and were growth-arrested by withdrawal of PDGF, which resulted in the entry of cells into G_0_; however, in contrast to our experiments, no significant increase in cells undergoing apoptosis was observed. This entry of cells into quiescence rather than apoptosis is consistent with the continued presence of low concentrations of serum and IGF-1 in the experiments of Coller *et al *[65], in contrast to the complete inhibition of PI 3-kinase in our studies. Comparing these two studies indicated that very little overlap exists between the genes regulated by inhibition of PI 3-kinase signaling and those regulated by 14 hours or 4 days of PDGF withdrawal. However, most of the overlapping genes, including *CCNG2, PLAU, BDNF*, and *FGF2*, and a number of non-overlapping genes regulated by mitogen withdrawal have been connected to the regulation of cell proliferation. Thus, there are functional overlaps in the genes regulated by PI 3-kinase inhibition and mitogen withdrawal, although they are clearly distinct gene sets as might be expected from the differences in treatments and their distinct effects on proliferation and apoptosis. We note that asynchronous cells were used in our study, and that similar analyses of synchronized cells might reveal PI 3-kinase regulation of distinct subsets or additional genes during specific stages of the cell cycle.

We have previously used computational analysis to identify transcription factor binding sites that are over-represented in promoter regions of immediate-early genes, including those regulated by PI 3-kinase signaling [26,30,44]. The transcription factors and families involved in regulation of these genes included CREB, SRF, FOXO and NFκB. We applied a similar analysis to the gene sets affected by inhibition of PI 3-kinase signaling in the present study. This revealed over-representation of phylogenetically conserved binding sites for FOXO and NFκB upstream of the genes that were up- and down-regulated, respectively, following 2 and 4 hours of PI 3-kinase inhibition. Many of the predicted FOXO and NFκB binding sites were confirmed by ChIP, indicating that the FOXO and NFκB transcription factors play major roles in the transcriptional response to PI 3-kinase inhibition. This is consistent with the established regulation of these transcription factors by PI 3-kinase/Akt signaling, which inhibits FOXO and promotes NFκB activity [16,19,20]. Since p53 is also a major target of PI 3-kinase/Akt signaling in control of cell proliferation and survival [12,13], we might have expected to similarly observe an over-representation of p53 binding sites. However, *p53 *is inactivated by mutation in T98G cells [66,67], accounting for its absence in the gene sets analyzed in our experiments.

In total, we identified FOXO and NFκB binding sites upstream of about one-third of the genes that were up- and down-regulated, respectively, by inhibition of PI 3-kinase. Since our computational predictions of transcription factor binding sites were limited to phylogenetically conserved sites in promoter regions, this may be an underestimate of the total fraction of PI 3-kinase regulated genes that are targeted by these transcription factors. In particular, additional genes may be regulated by FOXO or NFκB binding sites in enhancers rather than promoter sequences, or by sites that were not conserved in the mouse. Other genes may be regulated at the transcriptional level by other transcription factors (such as Myc/Max) or by non-transcriptional mechanisms, including regulation of mRNA decay [27].

Two members of the FOXO family (FOXO1 and 3a) were expressed in T98G cells and, as expected, both were activated in response to PI 3-kinase inhibition. In contrast, there are 5 members of the NFκB family (p65, c-Rel, RelB, p50 and p52), which can be activated by either the canonical or non-canonical pathways, both of which are stimulated by Akt [19,20,64,68-70]). We initially demonstrated binding of p65 to predicted NFκB sites following stimulation of the canonical pathway by TNFα. In unstimulated cells growing in the presence of serum however, only binding of p50, p52 and RelB was detected by ChIP, indicating that just the non-canonical pathway was active. Of these three family members, RelB is the main effector of this pathway, as it is the only one that contains a transactivation domain. Since the binding of RelB to promoters of down-regulated genes decreased following inhibition of PI 3-kinase, it appears that PI 3-kinase signaling regulates the non-canonical pathway of NFκB activation in proliferating cells.

The 10 genes identified as targets of FOXO and the 18 identified as targets of NFκB are summarized in Table 7. Binding sites for FOXO had been previously identified upstream of 4 of these genes, and new sites were identified upstream of 6 genes in the present study. Similarly, the 22 NFκB binding sites identified upstream of 18 genes included 10 that were previously known, 5 that were newly identified in genes previously known to be regulated by NFκB, and 7 upstream of new NFκB target genes identified in the present study. It is noteworthy that nearly half of the FOXO and NFκB target genes whose expression is altered in response to inhibition of PI 3-kinase are known to function as regulators of apoptosis, including the previously unrecognized FOXO targets *DDIT3/CHOP *and *TXNIP *and the NFκB targets *AMIGO2, BDNF, IGFBP5*, and *TNFRSF11B/osteoprotegerin*. The predominant functions of these PI 3-kinase regulated genes emphasize the critical role of PI 3-kinase/Akt regulation of the FOXO and NFκB transcription factors in maintaining cell survival.

**Table 7:** FOXO and NFκB regulated genes.

	Growth Factor/Receptor or Cytokine	Transcription	Proliferation	Apoptosis	References
**FOXO Target Genes**					

***ATROGIN-1***					[**51**]

***BCL6***		**+**		**+**	[**49, 50, **80]

***BTG1***			**+**		[**48, **81]

***CCNG2***			**+**		[41,**46, 47**]

*DDIT3/CHOP*		**+**	**+**	+	[36, 82]

*KIF1B*					

*KLF6*		**+**	**+**		[83]

*KLHL24*					

*TXNIP*				+	[84]

*ZBTB1*					

**NFκB Target Genes**					

*AMIGO2*				+	[85]

*BDNF*	**+**		**+**	+	[86]

***BIRC3/cIAP2***				**+**	[**54**, 87]

***CCL2***	**+**			**+**	[**55**, 88]

***CCND1***			**+**		[**56**]

***EDN1-site I***	**+**		**+**	**+**	[**57, **89, 90]

*EDN1-site II*	**+**		**+**	**+**	[89, 90]

*F3-site I*					

***F3-site II***					[**58**]

*FST*	**+**		**+**		[91]

*G0S2*			**+**		[92]

***GABPB2***					[**53**]

*IGFBP5*	**+**		**+**	**+**	[93, 94]

***IL7R-site II***	**+**		**+**	**+**	[**59**, 95]

*IL7R-site I*	**+**		**+**	**+**	[95]

*MAT2A-site I*					

***MAT2A-site II***					[**60**]

***PLAU***			**+**	**+**	[**61**, 96]

***PTX3***					[**62**]

*RGS20*					

*STC2*					

*TNFRSF11B/osteoprotegerin*				+	[40]

All PI 3-kinase regulated genes containing upstream binding sites for either FOXO or NFκB are listed. Genes containing a previously reported FOXO or NFκB binding site, and the relevant reference, are in bold. The biological activity of each gene, as it pertains to growth factor/receptor or cytokine, transcription, proliferation, and apoptosis, is indicated along with references for these activities.

## Conclusion

PI 3-kinase signaling in proliferating cells regulates a novel program of gene expression, which is distinct from that induced by growth factor stimulation of quiescent cells. The expression program controlled by continuous PI 3-kinase signaling in proliferating cells is enriched in genes that regulate cell survival and is mediated in large part by FOXO and RelB transcription factors.

## Methods

### Cell Culture and RNA Extraction

T98G human glioblastoma cells were grown in Minimal Essential Medium (Invitrogen) containing 10% fetal bovine serum (HyClone) and 100 units/ml of penicillin/streptomycin (Invitrogen). U937 cells were grown in RPMI 1640 (Cellgro) containing 10% fetal bovine serum (HyClone). For LY294002 treatments, T98G cells were plated at 2 × 10^6 ^cells per 150 mm plate or 8 × 10^5 ^cells per 100 mm plate and U937 cells at 3 × 10^6 ^cells per 25 cm flask. T98G cells were cultured for 48 hours and U937 cells for 24 hours, at which time they were actively proliferating with a doubling time of approximately 20 hours. LY294002 (Biomol) was added to a final concentration of 50 μM. Wortmannin (Biomol) was added to a final concentration of 50 nM. Culturing of cells for serum starvation and subsequent treatments with either PDGF-BB (50 ng/ml) or 20% serum were carried out as previously described [44].

### Apoptosis Assays

Apoptosis was assayed by DNA fragmentation and TUNEL staining as previously described [71].

### Immunoblot

Enriched cytosolic and nuclear fractions were isolated from normal growing T98G cells as described elsewhere [72]. Proteins were separated by electrophoresis in 8% or 12% SDS-polyacrylamide gels, electroblotted to polyvinylidene difluoride membranes (PerkinElmer), and immunoblotted with anti-phospho Akt (Cell Signaling 9271), pan-anti-Akt (Cell Signaling 9272), anti-FOXO3a (Upstate 07-702), anti-FOX01 (Santa Cruz sc-11350), anti-FOXO4 (Santa Cruz sc-5221), anti-PARP (poly (ADP-ribose) polymerase) (Cell Signaling 9542), and anti-14-3-3 (Upstate 06-511) as recommended by the manufacturer. The immunoblots were visualized using goat anti-rabbit or rabbit anti-goat horseradish peroxidase-linked secondary antibodies (Bio-Rad) and chemiluminescence (PerkinElmer). Densitometry was performed using IQMac v1.2 software (Molecular Dynamics, Sunnyvale, CA).

### Microarray Analysis

Microarray spotting, sample preparation, hybridization, image analysis and data analysis were performed as previously described [44]. Microarrays were spotted with 21,329 70-mer oligonucleotides from Operon's Human Genome Array-Ready Oligo Set Version 2.0. RNA for microarray experiments was extracted with TRIzol reagent (Invitrogen) followed by poly(A)+ RNA isolation with an Oligotex mRNA Midi Kit (Qiagen) according to each manufacturer's protocol. Microarrays were performed with three independent biological samples. Dye-swap, background-subtracted median intensity values were used as input to the LIMMA analysis package [73] in Bioconductor [74], and average LOESS-corrected log_2 _ratios were used to estimate differential gene expression after LY294002 treatment. Differentially expressed genes were those with a change in expression greater than or equal to 1.87 fold (Log_2 _0.9) relative to untreated samples and false discovery rate (FDR)-corrected [75] moderated *t*-test *p*-values less than 0.01.

### Real-time reverse transcription-polymerase chain reaction (RT-PCR)

Real-time RT-PCR was carried out as previously described on RNA samples isolated from either T98G cells or U937 cells treated with LY294002 for 4 hours [26].

### Gene Ontology Analysis

Gene Ontology (GO) terms were obtained using the web-based tool GOstat [76]. The genes that were either up- or down-regulated following 2 and 4 hours of PI 3-kinase inhibition were independently analyzed from the genes that were either up- or down-regulated following 8 hours of PI 3-kinase inhibition. Enrichment of GO categories was determined by comparing the terms associated with the differentially regulated genes to the terms associated with all genes on the array using a Fisher's exact test. Only genes annotated with at least 1 term were included in the analysis. Results were limited to the GO categories that were associated with at least 15% of the genes, with *p *< 0.001.

### Transcription Factor Binding Site Analysis

Over-representation of transcription factor binding sites in the upstream regions of genes that were differentially expressed in response to inhibition of PI 3-kinase was determined as previously described [26,30,44]. The genes that were either up- or down-regulated following 2 and 4 hours of PI 3-kinase inhibition were independently analyzed from the genes that were either up- or down-regulated following 8 hours of PI 3-kinase inhibition. The regions 3 kb upstream of transcription start sites in the human and the corresponding mouse orthologous sequences were analyzed with the Match program using the MinSUM threshold [77], and both the 548 vertebrate position weight matrices from TRANSFAC Professional version 8.4 and the 588 matrices from TRANSFAC version 11.1 [78]. Sequences and MULTIZ alignments were obtained from the University of California Santa Cruz Genome Browser (human version hg18, mouse version mm8) [79], which were available for 28 of the 32 up-regulated genes and for 48 of the 53 down-regulated genes. For each matrix, a permutation test was used to compare the frequencies of the predicted sites in the differentially expressed gene set to the frequencies of the predicted sites in a background set of 662 genes that were expressed but did not change expression upon PI 3-kinase inhibition (average log_2 _ratios between -0.01 and 0.01). *P*-values were FDR-corrected to adjust for multiple testing [75].

### Flag-FOXO3a-AAA Chromatin Immunoprecipitations

T98G cells were plated at 4 × 10^5 ^cells per 100 mm plate in complete media 24 hours before transfection. TransIT (Mirus Bio) reagent was used to transfect 8 μg of either pcDNA3 (Invitrogen) or pcDNA3-Flag-FOXO3a-AAA [Addgene plasmid 10709, provided by Dr. William R. Sellers [46]], as recommended by the manufacturer. 24 hours after transfection 3 identical plates were harvested and ChIP assays were performed as previously described [30] except that chromatin was immunoprecipitated overnight at 4°C using 10 μg of anti-M5-Flag (Sigma F4042) antibody and Protein G agarose beads (Upstate). Protein G agarose beads were washed successively in low salt wash, high salt wash, LiCl wash and twice in 1xTE. Immunoprecipitated chromatin was quantified with real-time PCR using primers designed within 250 bp of the predicted transcription factor binding sites.

### NFκB Chromatin Immunoprecipitations

For treatment with TNFα, T98G cells were plated at 8 × 10^5 ^cells per 100 mm plate in complete media 48 hours before treatment. Cells were then either left untreated, or treated with 20 μg/ml of TNFα (R & D Systems) for 0.25, 0.5 or 1 hour. For experiments using unstimulated cells, T98G cells were plated at 2 × 10^6 ^cells per 150 mm plate, and ChIP assays performed 48 hours after plating. When indicated, cells were treated with 50 μM LY294002 for 4 or 8 hours. ChIP assays were performed as previously described [30], except that either 5 μg of p65 antibody, c-Rel antibody, p50 antibody, RelB antibody (Santa Cruz Biotechnology, sc-372, sc-71, sc-114, sc-226), or 5 μl of p52 antibody (Upstate, 06-413) was used for the immunoprecipitations. Protein A agarose beads were washed successively in low salt wash, high salt wash, LiCl wash and twice in 1xTE. Immunoprecipitated chromatin was quantified by real-time PCR using primers that were located within 320 bp of the predicted binding site.

## Authors' contributions

JT and JRG contributed equally to all aspects of the study, JWT contributed to the overall study design and experimental analysis of FOXO activation, KWA to analysis of apoptosis following PI 3-kinase inhibition, MES to analysis of the microarray data, and GMC to conception and analysis of the study. JT, JRG, and GMC were primarily responsible for preparation of the manuscript, with critical comments and revisions from MES and JWT. All authors read and approved the final manuscript.

## Supplementary Material

Additional file 1 Expression changes arising after 2, 4, and 8 hours of PI 3-kinase inhibition Click here for file

Additional file 2 Effects of LY294002 and wortmannin on gene expression Click here for file

Additional file 3 TRANSFAC matrices tested for over-representation in the genes that were differentially expressed after 2 and 4 hours of PI 3-kinase inhibition Click here for file

Additional file 4 Predicted FOXO and NFκB binding sites in the genes that were either up- or down-regulated after PI 3-kinase inhibition Click here for file
