# Supplementary material for: Phosphatidylinositol 3-kinase signaling in proliferating cells maintains an anti-apoptotic transcriptional program mediated by inhibition of FOXO and non-canonical activation of NFκB transcription factors
Source: BMC Cell Biol. 2008 Jan 28;9:6. doi: 10.1186/1471-2121-9-6 (PMC2268685; doi:10.1186/1471-2121-9-6)

Additional File 2 - Effects of LY294002 and wortmannin on gene expression

Proliferating T98G cells were treated with either 50 µM LY294002 or 50 nM wortmannin for 4 hours. *A*, Gene expression was quantitated by RT-PCR. Data are expressed as average fold changes from two independent experiments. *B*, Whole cell extracts were subjected to SDS-PAGE and immunoblotted with antiphospho Akt. Extracts of untreated cells were diluted 1/5 and 1/10 for comparison with undiluted extracts of LY294002 and wortmannin treated cells

A

	Fold change	
	LY294002	Wortmannin
Up-regulated genes		
ATROGIN-1	3.5	3.6
BCL6	3.0	2.2
BTG1	8.2	4.1
CCNG2	2.4	3.0
DDIT3/CHOP	2.2	0.7
IRF7	3.6	1.2
SAT1	2.0	1.4
TXNIP	3.4	1.9
Down-regulated genes		
BIRC3/cIAP2	11.3	2.5
CCL2	17.1	1.8
CCND1	6.1	4.4
DCK	1.8	1.3
DSCR-1	2.8	2.1
EDN1	39.5	14.3
G0S2	7.8	3.4
PLAU	3.0	2.2
PTX3	5.6	4.6
TNFRSF11b/OPG	3.6	1.1

B

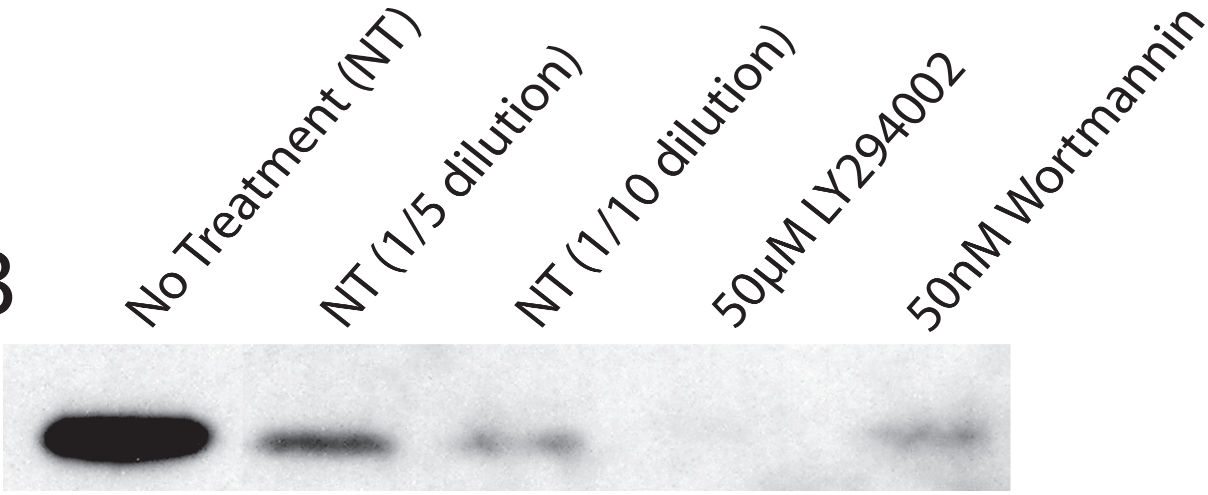

Supplement: Additional file 2 — Effects of LY294002 and wortmannin on gene expression [file 1471-2121-9-6-S2.pdf]
